# The Management of Uveitic Glaucoma in Children

**DOI:** 10.4274/tjo.galenos.2019.36589

**Published:** 2019-10-24

**Authors:** Dimitrios Kalogeropoulos, Christos Kalogeropoulos, Marilita M. Moschos, Velota Sung

**Affiliations:** 1 Birmingham and Midland Eye Centre, Sandwell and West Birmingham Hospitals NHS Trust, United Kingdom; 2 Department of Ophthalmology, Faculty of Medicine, School of Health Sciences, University of Ioannina, Ioannina, Greece; 3 First Department of Ophthalmology, General Hospital of Athens G. Gennimatas, Medical School, National and Kapodistrian University of Athens, Greece

**Keywords:** Glaucoma, uveitis, children

## Abstract

Children comprise a unique population of patients in regard to the diagnostic and therapeutic approach of uveitic glaucoma. The management of glaucoma secondary to uveitis in children is extremely challenging and presents various difficulties, which are associated both with the underlying uveitis and the young age of the patients. The treatment of uveitic glaucoma calls for a thorough and individualized approach, involving both pharmacotherapeutic and surgical modalities. It appears that the efficient control of inflammatory activity plays a significant role in the final visual outcome of these patients. This study aims to review the current literature about the management of uveitic glaucoma in pediatric patients.

## Introduction

The evaluation and management of uveitis in children is extremely challenging for the ophthalmologists that have to confront this clinical entity, whereas glaucoma in children is a potentially blinding condition. Uveitis can lead to several complications, such as secondary glaucoma, cataracts, synechiae, band keratopathy, and macular edema.^1^ There is some evidence that the rates of complications differ between adults and children, and some of the complications may be unique to children.^1^ Uveitic glaucoma represents a special category of secondary glaucoma in both adult and pediatric populations. The clinical outcomes of uveitic glaucoma in children depend on several factors (e.g., type, severity, and duration of the disease) and are often guarded, especially in complicated cases. The successful management of uveitic glaucoma in children calls for an early and accurate diagnosis and control of inflammation and intraocular pressure (IOP) to reduce the risk of progressive damage to the optic nerve and the risk of amblyopia.^2^ Treatment with ocular and systemic steroids, as well as with corticosteroid-sparing therapy has significantly contributed to the control of inflammation and improved the visual prognosis.^3^ In many cases, the successes of medical treatments are limited because of poor compliance or intolerable local or systemic side effects.^2^ Moreover, many uveitic patients with glaucoma may need surgical intervention to control IOP and preserve vision. There is high risk of significant visual loss from complications of uveitis and/or glaucoma over the lifespan of these patients, and this has significant impacts in terms of financial burdens, quality of life, and loss of productivity for the patients.^2^ This study focuses on the clinical features and management of uveitic glaucoma in childhood.

### Epidemiology

The overall annual incidence of uveitis among children in North America and Europe is lower compared to the rates for adults, which are approximately 4.3 to 6 in 100,000 population.^4,5^ The same epidemiological studies found that the prevalence of uveitis in childhood is roughly 30 cases in 100,000 population.^5^ The prevalence of pediatric glaucoma in uveitic patients varies between 5 and 13.5%.^6^ It has been reported that one third of these patients end up with poor vision due to the complications of uveitis. It appears that in children with glaucoma, uveitis may be the underlying cause at a percentage of 6 to 9%. According to the British Infantile and Childhood Glaucoma Eye Study, uveitis led to 19% of the glaucoma cases among 52 children with secondary glaucoma.^7^ Kaur et al.^2^ reported that among 385 children with glaucoma, 150 patients were diagnosed with acquired glaucoma but uveitis was the underlying cause in only 8 of them (5.3%). A previous study by Paroli et al.^8^ found that 25% of children with uveitis developed secondary glaucoma.

To our knowledge, there are many reviews and cases series about uveitis in childhood, but only a few prospective studies that highlight the specific issues of uveitis and uveitic glaucoma in children.

### Risk Factors

The eyes of children with uveitis seem to have an inherent predisposition to developing secondary glaucoma in comparison with adults.^2^ The underlying cause and the duration of the disease have been correlated with the prevalence of uveitic glaucoma. As mentioned above, the risk of developing uveitic glaucoma depends on the cause of uveitis, with higher incidences in Posner-Schlossman syndrome, uveitis associated with juvenile idiopathic arthritis (JIA), and herpetic infections.^9^ Interestingly, approximately 42 to 48% of the affected eyes, especially those with early onset glaucoma, are expected to have a poor visual outcome.^10^ It has been estimated that approximately half of cases with JIA-related glaucoma require surgical treatment for glaucoma.^11^

### Pathogenetic Mechanisms

Uveitic glaucoma can arise through either open or closed angle mechanisms. Secondary angle closure mechanism is commonly due to progressive peripheral anterior synechiae formation. Open angle mechanism is commonly due to obstruction of the trabecular meshwork by debris and inflammatory cells, and chronic remodeling of the trabecular meshwork and the Schlemm’s canal, causing increased resistance to aqueous outflow.^12^ Elevation of IOP has been attributed to a wide spectrum of inflammatory factors leading to increased resistance in the outflow pathways, which is often exacerbated by the required topical treatment with steroids.^5^ Topical and in some cases systemic steroids are commonly needed long-term for the control of inflammation. However, steroid-induced glaucoma may hinder IOP control through accumulation of extracellular matrices in the trabecular meshwork.^5,12^

It is important to underline that in patients with uveitic glaucoma there is also a higher propensity for postoperative hypotony due to the impairment of ciliary body functions caused by the chronic and relapsing nature of the intraocular inflammatory activity. As can be expected, inflammation is likely to be more pronounced in eyes of uveitic patients after intraocular surgery and this can lead to a rapid and undesirable subconjunctival scarring response. The application of antimetabolite to reduce this scarring process can further increase the risk of hypotony.^5,12^

However, the analysis of the pathogenesis of uveitic glaucoma is not within the scope of this review and the reader is referred to our recently published study that focuses on the pathophysiology of uveitic glaucoma.^12^

## Therapeutic Approach and Management

### 1. Pharmacotherapeutic Options for the Management of Uveitis

Corticosteroids have been the gold standard for the treatment of noninfectious types of uveitis. Over the last decade, there has been a trend of early and aggressive administration of immunomodulatory agents both for adults and children. The aim of this approach is to avoid the side effects of topical and systemic steroid treatments and prevent severe complications related to noninfectious uveitis.^2^ According to the more traditional approach, the ophthalmologist should wait until visual acuity begins to deteriorate or complications develop before starting the patient on immunomodulatory treatment. However, this strategy is not considered appropriate by the majority of uveitis specialists recently. An individualized approach for each patient is highly recommended in order to investigate the risk factors for developing complications and consider the option of selective immunomodulatory therapy.^2^ Children being treated with systemic steroids and/or immunosuppressive medication must be monitored very carefully, as these agents may affect their general health, growth, nutrition, school and other activities, or even their fertility.^13^

As can be expected, parents are concerned about how long uveitis will last and how long the course of treatment will be. It is important to explain to them that the course of the uveitis depends on the type and form of the disease. Some clinical entities (e.g., HLA-B27-associated uveitis, toxoplasmic retinochoroiditis) can completely resolve if the appropriate treatment is administered. On the other hand, some cases of idiopathic uveitis or uveitis associated with systemic disorders (e.g., Kawasaki disease, post-streptococcal syndrome) may be less aggressive and transient.^2^ However, when there are prominent and persistent signs of inflammation, ophthalmologists should be prepared to plan their therapeutic strategy accordingly and take into account the possible complications. Interestingly, JIA-related chronic anterior uveitis may become less severe over time but is likely to continue even in adult life, and it is not uncommon for a joint inflammation to subside in cases of persistent iridocyclitis.

### a. Antimetabolites

Methotrexate remains the most commonly used immunosuppressive medication in the pediatric population. It is considered to be safe, does not affect future fertility, and is well-tolerated and easily administered. It has been shown that treatment with methotrexate is significantly safer for long-term use in comparison with oral steroids. Methotrexate is known for its efficacy in the treatment of JIA-related joint inflammation in children and is believed to also be effective in cases of JIA-related uveitis and chronic idiopathic anterior uveitis in children.^14^ Although several studies have reported the use of methotrexate in children with uveitis, there is a lack of randomized controlled trials and only sparse specific articles with regard to the response rates.^14^ In the clinical setting it appears that methotrexate is effective in more than 60% of patients with chronic uveitis,^2^ a rate which is comparable to the response rates for arthritis.^14^ It is suggested that methotrexate doses need to be higher on a mass-adjusted dosage scheme in children as it is metabolized more quickly in pediatric than in adult patients. The usual oral dose is 10-30 mg/m^2^ once weekly.^15^ Absorption may vary among individuals and the option of subcutaneous injection of methotrexate can be considered before deducing that the agent is ineffective. Additionally, subcutaneous injections may be better tolerated than per oral use in children, which may cause sickness and irritable or upset stomach.

Currently there is lack of evidence and experience in the use of other antimetabolites (i.e., azathioprine, leflunomide, and mycophenolate mofetil) in children with uveitis. However, mycophenolate mofetil has been reported as an alternative to methotrexate in cases of intolerance in children.^2^

### b. Cyclosporine and Cytotoxic Agents

Cyclosporine has been successfully used in several forms of uveitis in pediatric patients.^16^ Cyclosporine is generally administered to children with uveitis in the same dosage range (3-5 mg/kg daily) used in adults and is considered to be safe as it does not affect growth or gonadal function.^2^

Chlorambucil cyclophosphamide has been utilized in the treatment of various diseases such amyloidosis, Behçet’s disease, severe systemic lupus erythematosus, and other vasculitis. Due to the little experience in their use for the treatment of ocular inflammation and the potential of long-term side effects, their use is not suggested for non-life-threatening diseases.^2^

### c. Biologic Agents

Cytokine inhibitors have been reported to achieve significant success rates in several types of arthritis and other inflammatory diseases (e.g., Crohn’s disease) in children and therefore their use has been seriously considered for the treatment of uveitis as well.^2^ Until recently, etanercept (Enbrel, Immunex Corporation, Seattle, Washington, USA) and infliximab (Remicade, Centocor, Inc., Malvern, Pennsylvania, USA), which inhibit tumor necrosis factor (TNF), were the two main biological agents evaluated for patients with uveitis. More specifically, etanercept is a fusion protein that contains a portion of TNF receptor and binds with TNF, prohibiting the activation of cells. On the other hand, infliximab is a monoclonal antibody against TNF.^2^

However, these two agents have different mechanisms and it has not been yet determined which is more effective in children with uveitis. Apart from that, these drugs may affect various types of uveitis in different ways. It should be noted that infliximab is known to have a higher rate of complications, including development of antibodies to the agent and increased risk of tuberculosis.^2^ Moreover, it is not always easy to predict the long-term effects of drugs that target only one factor in the whole complex dynamics of the human immune system and the perplexity of immune responses. On the other hand, a human monoclonal antibody, the anti-TNF agent adalimumab (already administered for noninfectious intermediate and posterior uveitis and panuveitis), has also been recently approved for the treatment of pediatric chronic noninfectious anterior uveitis.^17^

More recent studies showed that of the TNF-α inhibitors, adalimumab and infliximab are the most effective in the control of ocular inflammation.^18^ More specifically, 87% and 72% of children with autoimmune chronic uveitis responded to adalimumab and infliximab, respectively, whereas they were refractory to other disease-modifying medications.^18^ One of the main advantages of adalimumab is that it can be administered subcutaneously at home (fortnightly), presenting more stable serum concentrations and a favorable safety profile with reduced risk of anaphylactic reaction. It has been recently demonstrated that the combined treatment of adalimumab with methotrexate is effective in cases of JIA-related uveitis. Interestingly, this study showed that a substantially larger proportion of children treated with adalimumab had reduced topical steroid dose or even discontinued topical steroids in comparison with the placebo group.^19^ Nevertheless, these patients had an increased incidence of adverse effects, including minor infections and gastrointestinal or respiratory disorders.

In cases that do not respond to TNF-α inhibitors, other biologic agents such as rituximab, interferon, or intravenous immunoglobulin can be alternatively administered. At present though, there are insufficient with regard to their use. Subsequently, clinicians should weigh the potential risks (e.g., malignancies, demyelinating disease, opportunistic infections) and benefits before proceeding to these therapeutic approaches.^20^

### d. Nonsteroidal Anti-inflammatory Drugs (NSAIDS)

Oral NSAIDS play a critical role in the management of joint inflammation in individuals with JIA. They have been deployed as an adjunctive therapy to other anti-inflammatory drugs in the therapeutic schemes in children with uveitis.^16^ However, their true benefit is yet to be confirmed as there is still inadequate evidence of their effect for this particular indication.^13^ Despite the demonstration of a link between NSAIDS and improved visual outcomes in patients with JIA-related uveitis, a causal relationship could not be proved. According to our experience and regarding the route of administration, the topical use (eye drops) of NSAIDs may contribute to the improvement of intraocular inflammation. Systemic NSAIDs can also be used while tapering the corticosteroids in order to avoid recurrence of inflammation, but some believe that their efficacy is also. It is also important to consider that the oral use of NSAIDs has been associated with gastrointestinal irritation, skin rashes, renal toxicity, and central nervous system rections.^21^

### 2. Pharmacotherapeutic Options for the Management of Glaucoma

Medical management of uveitic glaucoma in children is challenging and depends on the etiology, the patient’s age at presentation and general health, and the known efficacy and safety profiles of each drug. The main targets of medical treatment for pediatric glaucoma should be to achieve target IOP while maximizing compliance and minimizing side effects. A relatively wide spectrum of antiglaucoma medications is commercially available for the control of IOP but of these, only latanoprost has been officially licensed by the regulatory agencies for use in children.^22^ The range of drugs includes b-blockers, prostaglandin (PG) analogs, a-adrenergic agonists, topical and systemic carbonic anhydrase inhibitors, parasympathomimetics, and combined preparations. Timolol and PG are usually prescribed as monotherapy, and generally provide good diurnal control of IOP. With respect to combined preparations, the combination of timolol and dorzolamide is the most preferred therapy. Interestingly, most drugs have been found to have comparable ocular hypotensive effects, with the lowest occurrence of systemic side effects reported with PG analogs. Brimonidine is not that favored by the vast majority of pediatric ophthalmologists due to the potentially life-threatening side effects reported in infants. It is known to cause syncope in children as it crosses the blood-brain barrier easily in children. It is always important to take into account that systemic absorption of topical drops may have a greater impact in infants than in adults, leading to higher plasma levels for a longer period and eventually to a higher risk of serious systemic adverse effects. Moreover, the use of beta-blockers in children can cause unpleasant side effects such as asthma attacks, nightmares, and night-wetting.^23^ Some ocular adverse events related to latanoprost have been reported in adults, including ocular surface disorders and irritation, periocular skin pigmentation, cystoid macular edema, anterior uveitis, and reactivation of herpes simplex keratitis due to potential excitation of inflammation.^24^ Cytomegalovirus anterior uveitis has been also described in two immunocompetent individuals following travoprost and latanoprost eye drops.^25^ However, patients of this cohort were also on immunomodulatory therapy and therefore it is difficult to draw firm conclusions. Moreover, clinicians should keep in mind that parasympathomimetics (miotics) should be avoided in children with uveitic glaucoma.^26^ Other events of the ocular surface, such as irritation, itching, dry eye, sensation, blurred vision, allergy, blepharitis, and discharge can occur at a similar incidence with the use of all PG analogs.^22^ Despite controversies, some authors suggest that PG analogs and prostamides may be first-line therapy in patients with uveitic glaucoma,^27^ but only in cases of quiescent uveitis without previous complicated intraocular surgery or pre-existing CME and in eyes without a history of herpetic keratitis or keratouveitis. However, several clinicians are cautious with regard to the administration of those medications even in cases of quiescent uveitis. In every case, it is always necessary to check regional regulations with respect to off-label use and licensure of topical antiglaucoma drugs for children, as this can vary from one region to another. For detailed information about the available pharmacotherapeutic options for pediatric glaucoma, including their safety considerations and efficacy data, the reader is referred to the studies of Samant et al.^22^ and Chang et al.^26^

At the moment, evidence obtained from randomized controlled trials in children remains inadequate and we lack of knowledge especially in regard to the use of newer antiglaucoma preparations. Therefore, existing medical therapies for glaucoma need further and thorough evaluation in well-designed randomized control trials with pediatric populations in terms of their safety, effectiveness, and effect on central corneal thickness, ocular surface stability, and effectiveness of PF preparations.

## Surgical Treament of Glaucoma

### Special Considerations

Surgical treatment is one of the greatest challenges in the field of uveitic glaucoma and its successful management. Some of the difficulties that present derive from the inflammatory status, the legacy of previous surgeries,^27^ and intraocular sequelae secondary to complications from chronic uveitis. Most glaucoma procedures involve creating an alternative passage for aqueous humor drainage (e.g., trabeculectomy or glaucoma drainage device [GDD]).^27^ The aggressive healing response is considered to play a critical role in the lower surgical success rates in children compared to those of adults in these procedures. Therefore, trabeculectomy is often combined with the use of anti-scarring agents, though these may be associated with significant complications.^27^ The limited ability for children to cooperate and cope with the intensive postoperative topical drop therapy adds to the complexity of the situation. Caregivers have to overcome these difficulties and achieve cooperation with the children. Resistance from school and activity restrictions are not uncommon, whereas sometimes other caregivers may need to provide assistance while instilling the postoperative eye drops. A commitment to frequent visits to the eye hospital for postoperative follow-up is necessary, but this may affect the child’s school attendance and the carer’s work commitments.^27^

Postoperative manipulations (e.g., suture removal) and in some cases the clinical examination (especially in younger children) present two more obstacles that ophthalmologists need to overcome. For this purpose, examination under anesthesia may be required more than once.^27^ Generally speaking, uveitic glaucoma in children occurs in the older age group, usually after 5-7 year of age, therefore most will be cooperative in clinical examination. Examination under anesthesia is rarely performed for children with uveitic glaucoma unless there are associated learning disabilities. Moreover, concurrent ametropia correction and amblyopia therapy, when required, is necessary to achieve a favorable long-term visual outcome. It is noteworthy that amblyopia is not commonly seen due to uveitic glaucoma as most visual difficulties come after the sensitive period. Many visual disabilities are secondary to cataracts, glaucoma, band keratopathy, and then phthisis.

### Making the “Right” Choice

In regard to surgical approach, the success of controlling IOP and maintaining vision depends on the primary surgical approach (i.e., the first operation chosen) and developing a long-term surgical strategy.^27^ In uveitic glaucoma, the optimal surgical procedure calls for more than setting an algorithm, as the clinician has to consider the age of the patient, their general health, the underlying cause, past ophthalmic history (including previous ocular surgeries), the possibilities of further ophthalmic interventions (e.g., cataract extraction), the degree of glaucomatous optic nerve damage, and visual prognosis. Additionally, important factors such as family and social conditions (e.g., likelihood of follow-up, availability of carers, etc.), as well as the local facilities, cannot be ignored.^27^

Every ophthalmic surgeon needs to devote sufficient time discussing the potential risks and benefits of any surgical treatment with the parents, especially in refractory cases when the fellow eye is stronger and healthier or if the child has only eye.^27^

Generally, the main surgical options can be divided into the following categories:

1. Glaucoma drainage device (GDD), 

2. Trabeculectomy, 

3. Angle surgery, 

4. Minimally-invasive glaucoma surgeries (MIGS),

5. Cyclodestructive procedures (to reduce aqueous humor production).

### 1. Glaucoma Drainage Device

The first use of GDDs in pediatric patients was described by Molteno in 1973. Various GDDs were later introduced, but the Ahmed implant (96 mm^2^/184 mm^2^) (New World Medical Inc., Rancho Cucamonga, CA, USA) and the Baerveldt implant (250 mm^2^ and 350 mm^2^) (AMO Inc., Abbott Park, IL, USA) are the most commonly used.^28^ It is difficult to compare the success rates of GDDs among published studies, but all of them report reduced success over time and the need for adjunctive medication as two of the main common features. Although the success rate has been reported as approximately 80% at 1 to 2 years of follow-up,^7^ it drops around 50% in the longer term.^29^ It is hard to determine which GDD is the best choice for children and it appears that none of them is clearly superior. However, it has been reported that the Baerveldt implant may achieve better long-term IOP control, whereas the Ahmed implant may provide fewer short-term complications.^30^ Choice of implant depends on several factors, including the diagnosis, positioning, the surgeon’s experience, the child’s condition, and of course implant availability and affordability. Interestingly, GDD has been proposed as the primary surgery for children with uveitis (either aphakic or pseudophakic), children who develop glaucoma after cataract extraction, or those that will require cataract extraction in the near future.^27^

One of the most common and sight-threatening complications of GDD surgeries in children is postoperative hypotony. The risk may be mitigated by reducing aqueous flow with external ligation of both flow-restricted and unrestricted implants.^31^ Absorbable-suture external ligation is commonly used for non-valved implants to restrict the amount of aqueous outflow in the early postoperative period. The suture should open spontaneously at 6-7 weeks, therefore delaying aqueous drainage onto the footplate and reducing the risk of encapsulation of the tube footplate.^27^ Furthermore, unrestricted implants can be restricted by an intraluminal stent suture, which may be used in addition to reduce aqueous outflow and avoid hypotony when the external ligature dissolves in 6-7 weeks’ time. Despite these measures, hypotony remains a possibility unless a watertight tunnel into the anterior chamber is achieved. Otherwise, it has been suggested that hypotony may be minimized by implantation of GDD in a two-stage procedure (securing the plate to the sclera in the first stage and inserting the plate when encapsulation has been achieved as the second stage).^32^ However, surgeons rarely use a two-stage procedure nowadays.

Other than hypotony, a wide range of complications may occur, including lens touch with cataract formation, corneal touch with corneal decompensation, and iris touch with persistent iritis or dyscoria. Moreover, tube migration either into the anterior chamber or posteriorly out of the anterior chamber has been documented. Tube obstruction from iris, vitreous, hemorrhage, and fibrinous or inflammatory membrane can also occur, whereas tube erosion and exposure can lead to infections and endophthalmitis. In some cases, GDDs may cause cosmetic or eye motility issues. The Baerveldt 250-mm^2^ implant is mostly preferred for uveitic eyes, which are prone to aqueous hyposecretion, or microphthalmic eyes with smaller anterior chamber.^27^

For children with good immunomodulatory control of inflammation and appropriate follow-up, Ahmed valve implantation can be an effective and safe procedure for treating pediatric uveitic glaucoma, providing immediate IOP reduction. However, there is evidence that early drainage can lead to early bleb encapsulation over the footplate and patients may experience a hypertensive phase that requires early re-introduction of glaucoma medications.^27^

Tube exposure is an important complication in the long term. Differential diagnosis between uveitis relapse and endophthalmitis is important in patients who received GDD implantation. The incidence of endophthalmitis in GDD was reported at about 6% in the pediatric group in a single-center study by Mandalos and Sung.^33^ Early recognition of endophthalmitis is extremely important, and most will require emergency removal of tube and plate in order to prevent further proliferation of the infection, which can lead to total loss of sight.

GDD can reduce endothelial cell density over time, leading to corneal decompensation.^33^ Although there is no statistical difference, adults are expected to develop corneal decompensation more frequently than pediatric patients. However, there is no direct comparison of corneal decompensation rates between adults and children in the current literature. Chronic uveitis can also reduce endothelial cell count over time; therefore, adding a GDD to the microenvironment of the anterior chamber may increase the risk of corneal decompensation in uveitic glaucoma patients.^34^ Therefore, tube placement is paramount in patients with chronic uveitis. Routine shortening of the tube after stabilizing eye pressure may reduce the risks of future corneal complications, which is a strategy employed by one of the co-authors of this paper.

Over the years, GDD implantation techniques have changed greatly to improve the safety profile of this procedure. The use of intraluminal suture (e.g., 3/0 Supramid suture) in non-valved GDD and external ligature (6/0 vicryl suture) to delay drainage have improved the safety profile of GDD implants. The risk of complications has been reduced with other changes such as making small entries with 25-gauge needle to prevent entry site leakage, and fenestration of the extraocular portion of the tube (Sherwood slit) proximal to the external ligature with dissolvable suture to avoid excessive intraocular pressure elevation. The use of mitomycin C with GDD is more controversial and there is only anecdotal evidence that its use can enhance the long-term success of GDD,^35^ but high-dose mitomycin C (MMC) can increase the risks of profound hypotony and related serious complications. There have also been improvements in the management of postoperative hypotony, which uveitis patients are at much higher risk of. Chiam et al.^36^ reported the use of fixed-volume (0.1 mL) viscoelastic (Healon GV) may be an effective and safe method to resolve acute hypotony after the dissolution of the external ligature, but repeat injections were necessary in most cases.

Regarding the risk of tube exposure, apart from utilizing a patch graft material (i.e., sclera), it is advised that the tunnel should be created at least 1-2 mm from the limbus, although various techniques have been described.^25,27,28^ Posterior insertion through the pars plana or ciliary sulcus may be considered when there is a high risk of corneal decompensation or if the anterior chamber is very shallow, especially for pseudophakic or aphakic eyes. However, the higher incidence of complications (e.g., choroidal effusions, retinal detachment, etc.) with this approach cannot be ignored.

Mandalos and Sung^33^ investigated the outcomes and complications of GDD surgery in both children and adults and underlined how important it is for the surgeon to remain vigilant for postoperative complications. Ophthalmologists need to be alert for signs of bleb encapsulation or endophthalmitis in pediatric patients.^33^ Fibrosis and encapsulation around the plate remain the main reasons of GDD failure. In contrast with trabeculectomy, the advantage of reducing fibrosis with anti-scarring agents has not been established in GDD surgeries in pediatric eyes. However, some authors support that after failed GDD surgery, repeat GDD surgery with MMC may be successful. After GDD failure, the introduction of topical glaucoma medication is considered to be the simplest and lowest risk option. Alternatively, needling or surgical revision of the bleb over the plate (capsule excision) can be considered.^33^

Overall, surgical techniques have improved over time, leading to increased success rates and fewer complications. At present, the insertion of a glaucoma drainage tube seems to be the most promising surgical option, providing sufficient and long-term IOP control in children with secondary glaucoma.^22^

### 2. Trabeculectomy

Although the success rates in children have been shown to be much lower than in adults, this procedure is still probably one of the commonest first-line surgical treatments for children with uncontrolled uveitic glaucoma. The first published studies on trabeculectomy in children presented results of eyes with very advanced glaucoma and several previous surgeries. As expected, the results were poor and the complication rates were high.^37^ MMC, which is a potent inhibitor of fibroblast function, has been used to improve success rates. However, MMC can be used at various potencies requiring only intraoperative exposure, which is a great advantage over 5-fluorouracil (5FU) in pediatric patients. MMC is suggested for eye surgeons experienced in its use,^27^ as it has been correlated with higher complication rates, including early complications associated with hypotony (i.e., shallow or flat anterior chamber, choroidal effusion, hypotony maculopathy, suprachoroidal hemorrhage) and late complications related to thin, avascular, cystic blebs, which are generally more prone to leakage and potentially blinding infection. Due to these potential complications, trabeculectomy is a challenging procedure in pediatric glaucoma. Nevertheless, other authors^39^ reported that according to their experience and by suitable modifications to the surgical technique in combination with appropriate anti-scarring potency and its application technique (Moorfields Safer Surgery System),^27^ trabeculectomy can lead to satisfactory outcome in most cases. An anterior chamber maintainer can be used in these cases, not only for minimizing intraoperative hypotony, but for enabling the precise judgment of flow through the scleral flap.^27^ With the modified technique, many glaucoma specialists consider trabeculectomy to be the first-line procedure for the majority of secondary glaucomas in children with the exception of those known to have a poor prognosis, such as aphakic or pseudophakic eyes associated with uveitis. Similarly, in other secondary glaucomas, the presence of cataract or corneal disorder that may soon require lens extraction or corneal transplantation, respectively, should be considered contraindications for trabeculectomy.^27^

A more recent study by Wang et al.^38^ evaluating trabeculectomy outcomes in 33 pediatric patients with uveitic glaucoma showed that IOP control improved and the number of anti-glaucoma medications decreased without any major complications. Additionally, visual acuity and intraocular inflammation remained stable (p>0.05), suggesting that trabeculectomy is safe and effective for these patients. The suitability of trabeculectomy specifically in JIA-related uveitic glaucoma was highlighted in a retrospective study of 21 children showing good IOP control and an overall success rate (with topical anti-glaucoma medication) of 71.4% after 5 years.^39^ Leinonen et al.^40^ examined the results of the potential effect of treatment with systemic tumor necrosis factor (TNF) inhibitor on the success of an MMC-augmented trabeculectomy for individuals with JIA-related uveitic glaucoma. They reported that trabeculectomy success rates at 1, 5, and 10 years after surgery were higher among patients treated with TNF inhibitors (at the time of their trabeculectomy to control uveitis, arthritis, or both) when compared with those who were not treated with TNF inhibitors.

In cases where trabeculectomy fails to control IOP, bleb needling with an anti-scarring agent may be required, but only if the bleb architecture allows this intervention and the sclerostomy is patent. It is noteworthy that needling may be necessary with early failure. Repeat trabeculectomy with a stronger dose of MMC may be required. Otherwise, a GDD can be considered if further surgery is needed.^41^

### 3. Angle Surgery

The main concept of angle surgery is to control the innate or “natural” outflow mechanism by facilitating aqueous access to Schlemm’s canal and the collector channels. Originally, this approach was deployed as primary surgery for primary congenital glaucoma (PCG), but some have supported its use in some types of secondary glaucoma, such as juvenile open-angle glaucoma (JOAG) or aphakic glaucoma. However, it is regarded as less successful except for uveitic glaucoma and in cases with ‘PCG-like’ angles, such as in congenital rubella or infantile presentations of Sturge-Weber syndrome.^27^

Goniotomy provides an internal approach through a paracentesis and trabeculotomy from an external approach using a scleral cut down to access Schlemm’s canal.^42^ Since its introduction, goniotomy has undergone only minor modifications, such as using needles instead of a tapered knife for the angle incision, several goniolenses, viscoelastic or anterior chamber infusion for maintaining anterior chamber depth, and hyperosmotic solutions to clear the epithelial edema. However, the procedure can provide satisfactory IOP control in many cases of uveitic glaucoma and has several advantages over GDD surgery in these patients, including shorter operative time and preservation of conjunctiva for future procedures. Moreover, re-operation is not necessary in many cases, as it may be with GDDs (e.g., tube exposure, suture removal, etc.).^41^ During goniotomy, an endoscopic approach enables visualization of the angle in conditions of poor corneal clarity, but this technique has not been adequately studied. Goniotomy represents a fairly successful and low-risk surgical treatment for uveitic glaucoma in children. However, it must be underlined that not all eyes (e.g., aphakic eyes with peripheral anterior synechiae) are ideal for angle surgery. Overall, goniosurgery is considered to be generally successful in children with glaucoma secondary to uveitis.

On the other hand, the trabeculotomy technique of angle incision has been modified, from the trabeculotome (which is a metal probe) to a blunted suture filament or an illuminated microcatheter (which allows visualization of its passage via Schlemm’s canal) that potentially facilitates the treatment of the whole angle in one surgery.^43^ The main debate with respect to the choice between goniotomy and trabeculotomy was based on the potential impact of the chosen procedure on future glaucoma surgical procedures and corneal clarity. However, the number of relevant randomized controlled trial studies among these two approaches is small. In general, the success rates of these two procedures are similar.^44^ Those who advocate goniotomy do so on the basis of the long-term effects in pediatric patients who are likely to undergo further glaucoma surgery at some point in their life. The long-term success of trabeculotomy is uncertain when there have been previous surgeries involving the conjunctiva, as the scleral cut-down distorts the conjunctiva and sclera, making a future trabeculectomy challenging and prone to failure. A temporal approach could preserve the superior site for future trabeculectomy.^41^

Goniotomy and trabeculotomy have been widely used since their introduction and their difference has to do with the approach to the angle. The main advantage of trabeculotomy over goniotomy is the ability to access potentially 360^o^ of the angle and the fact that it can be carried out even in eyes with opaque cornea. Even in cases with corneal haziness attributed to epithelial microcystic edema, special maneuvers can be performed in order to achieve adequate corneal clarity and achieve a favorable goniotomy. Both goniotomy and trabeculotomy (less than 360°) with probes can be repeated in cases of insufficient response.^41,44^

Goniosynechialysis (GSL) is another alternative for angle surgery. Initially described by Campbell and Vela in 1984, GSL is a surgical technique that aims to strip the peripheral anterior synechiae (PAS) from the trabecular surface in the angle and make a renewed pathway for aqueous to the trabecular meshwork. The procedure can be performed using an iris spatula, a cyclodialysis spatula, an Ahmed micro-grasper, or a bent 25-gauge needle to manually release the PAS. GSL appears to be effective for the treatment of chronic angle closure glaucoma and has been described as a combined technique with phacoemulsification.^45,46^ GSL could potentially be considered in some patients with glaucoma secondary to uveitis in order to resolve the PAS and improve trabecular outflow. However, there are limited data to support the use of this surgical modality in pediatric uveitic glaucoma.

If IOP is still not acceptable after angle surgery, filtration surgery can be considered as the next step. Unfortunately, especially for uveitic glaucoma, there are still not adequate randomized control trials to define the optimal primary surgical treatment. Most surgeons usually perform trabeculectomy surgery after angle surgery fails.

### 4. Minimally Invasive Glaucoma Surgeries

Over the last few years, there has been an increasing interest in the development of new devices and surgical techniques for MIGS. At the moment there is still not a widely accepted definition of MIGS.^46^ The term MIGS comprises a group of surgical procedures which are defined by five basic characteristics: (1) an ab interno approach via a clear corneal incision, (2) a minimally traumatic technique to the target tissue, (3) a justified approach based on IOP lowering efficacy, (4) a high safety profile with low rate of complications compared to other surgical modalities, and (5) a quick recovery taking into account the patient’s quality of life. In February 2014, during a workshop of the American Glaucoma Society and US Food and Drug Administration (FDA), MIGS was described as the insertion of a surgical device in order to lower IOP through an outflow mechanism with either an ab interno or ab externo approach, associated with minimal or no scleral dissection.^47^

As a matter of fact, many of these devices do not require a scleral incision and can be implanted ab interno via a clear corneal incision. Therefore, these procedures are frequently combined with phacoemulsification and intraocular lens (IOL) implantation. The main target of MIGS is to achieve a lower IOP with shorter operative times, and ideally accompanied by a medication-sparing effect. This is accomplished by increasing the outflow of aqueous humor from the anterior chamber by (i) directly accessing Schlemm’s canal, (ii) shunting aqueous humor to the suprachoroidal, or (iii) shunting aqueous humor to the subconjunctival space.^46,47^

In conventional glaucoma surgery (e.g., trabeculectomy), potential complications include bleb infection/inflammation, hyphema, hypotony, bleb revision, and endophthalmitis, and may occur in up to 35% of patients. These complications may be avoided with MIGS, offering an important therapeutic alternative in individuals with glaucoma. However, it is important to underline that efficacy and the incidence of complications and adverse effects may vary among the different types of MIGS procedures.^46,47^

The first three devices, iStent, iStent inject (Glaukos Inc., Laguna Hills, CA, USA), and Hydrus (Ivantis Inc., Irvine, CA, USA) aim to increase trabecular outflow by targeting the juxtacanalicular area of the trabecular meshwork, which likely represents the greatest resistance to aqueous humor outflow in eyes with OAG.^46,47^ These devices provide more direct access of aqueous humor from the anterior chamber into Schlemm’s canal. However, this approach does not allow postoperative IOP to decrease below the episcleral venous pressure (EVP), which may be increased in some glaucomatous patients.^46,47^ On the other hand, the CyPass micro-stent (Alcon Inc., Fort Worth, TX, USA) and iStent^®^ Supra aim to create an outflow pathway from the anterior chamber to the supraciliary space.^46^ Finally, the surgical concept of the XEN gel stent is to create a non-physiological route for the outflow of aqueous humor via subconjunctival filtration. This pathway is actually the basis for conventional trabeculectomy and for glaucoma epibulbar shunt surgeries.^46^

Apart from the implantation of the micro-stents mentioned above, MIGS includes the following more surgical techniques: trabectome, gonioscopy-assisted transluminal trabeculotomy, excimer laser trabeculotomy (increase of trabecular outflow), and endocyclophotocoagulation (reduction of aqueous production).

MIGS devices can lower IOP but the efficacy of these new surgical modalities, especially in childhood, needs to be confirmed by more studies. Existing studies have several limitations, including their retrospective nature, lack of standardization, lack of knowledge about the IOP lowering effect, concomitant use of more than one surgical procedure (e.g., phacoemulsification/IOL implantation and micro-stents) and inadequate information about ideal patient selection for these therapeutic tools.^46^

The concomitant application of various treatments and glaucoma devices in clinical studies, together with the variable populations and diverse study designs make it more difficult to evaluate and compare the final outcomes.

### 5. Cyclodestructive Procedures

The aim of cyclodestruction is to reduce aqueous humor production by using cyclocryotherapy, which has been associated with major complications and poor long-term outcomes in children. It is generally only reserved for selected challenging refractory cases. Over the course of time it was replaced with laser cyclophotocoagulation, which is a less destructive technique. More specifically, transscleral diode laser (810 nm) gained popularity over Nd:YAG laser.^48^ Transscleral diode laser is better tolerated and causes fewer complications. The ciliary processes can be precisely treated with endoscopic diode laser,^51^ but this requires an intraocular approach and caution when it comes to phakic eyes. Possible complications of diode laser include conjunctival burns, uveitis, hypotony, scleral perforation, cataract, retinal detachment, loss of vision, and phthisis.^48^ It is suggested that transscleral diode laser can be used together with transillumination of the eye in order to enhance laser accuracy and ensure better placement, avoiding scleral thinning, hemorrhage, or areas of pigmentation. Transscleral diode laser can be used in painful and blind eyes or in eyes with poor visual potential. Other indications include surgery with poor prognosis (that may be difficult or impossible), severe scarring of conjunctiva, or other ophthalmic abnormalities that may be present after filtrating surgeries.^27^ It has been reported that short-to-midterm success rates of transscleral diode laser are over 50%, but the high retreatment rate and the continuation of medication must be taken into account. The success rates of endoscopic diode laser have been found to be similar.^49^

The use of cyclodiode laser is not advised in children with uveitis, as it aims to reduce ciliary body function, which may already be compromised due to the inflammatory process and has generally been correlated to poor outcomes in this patient group. Additionally, a future, more invasive surgery can potentially lead to severe issues related to chronic hypotony.^27^

## Discussion

In the past, patients with uveitic glaucoma had poor visual outcome due to delayed diagnosis and the limited anti-inflammatory and antiglaucoma therapeutic options.^1,2^ Over the last two decades, advances in diagnostic tools and new systemic anti-inflammatory medications have provided clinicians with more sophisticated approaches that can prevent late consequences of uveitis.^1^ However, uveitis remains a potentially devastating condition that can have severe impacts on vision through various complications such as glaucoma, cataract formation, macular edema, and formation of synechiae.^3^ More specifically, cataracts are very often associated with uveitis, either directly due to the inflammation or indirectly due to the use of topical and oral steroids. In eyes with chronic inflammation activity, cataract extraction can cause an exuberant postoperative inflammatory reaction, which can lead to complications including glaucoma, hypotony, macular edema, and optic disc swelling.^50^

In young children, regardless of whether reduced visual acuity derives from glaucoma, uncontrolled inflammation, or other complications, it can lead to amblyopia and consequently to life-long visual disability. This is also expected to affect the child’s education and performance at school. Early, prompt, and efficient management of uveitic glaucoma is significant, especially in patients of amblyogenic age (i.e., younger than 7-8 years old).^21^ Amblyopia should be treated with occlusion therapy, and when the issue is resolved and the eye is not inflamed, the child can have a refraction test for optimizing visual function. Furthermore, in children that have gone through postoperative aphakic rehabilitation, the presence of a specialist pediatric contact lens optometrist would be more than helpful.^21^

The treatment of glaucoma secondary to uveitis has several challenges, especially when it comes to surgical intervention. One of the major issues is the fact that in many cases there is an intense inflammatory reaction, which complicates both the control of uveitis and eye pressure.^41^ The administration of topical and periocular steroids has been correlated with high risk of several ocular complications in children. IOP elevation and steroid-induced glaucoma in particular can develop rapidly in children, become refractory to treatment, and persist even after stopping topical corticosteroids. Likewise in the adult population, systemic corticosteroids should be used mainly for limited periods due to the wide spectrum of adverse systemic effects. Moreover, systemic steroids can cause adverse ocular effects including glaucoma, cataract, and retinal and choroidal emboli.^1,2^ Additionally, when it comes to deciding the most suitable surgical intervention in those patients, it is important to take into account the status of the angle (i.e., whether the angle is open and the extent of synechiae formation). Ophthalmic surgeons should have a strategy that will offer the maximal chances of preserving vision and IOP over the long term with minimal ocular damage.^41^

Holistic management is one of the cornerstones of a successful approach to pediatric glaucoma. The management of this vulnerable group of patients calls for the expertise and collaboration of a multidisciplinary team. It is vital for the ophthalmologist to be in direct and continuous communication with the pediatricians and rheumatologists in order to ensure a thorough investigation for underlying systemic diseases and prompt initiation of disease-modifying agents if required. Before the administration of systemic medications, clinicians and pharmacists need to check that any kind of immunomodulatory was prescribed only if laboratory investigations were within normal limits.^21^ A pediatric glaucoma or uveitis nurse specialist could play a critical role in the training of patients and family in the administration of medications, especially when it comes to subcutaneous drugs.

Adequate monitoring of the uveitic glaucoma and response to treatment is crucial in children. Special attention should be paid to visual acuity and any changes in vision in children at risk for amblyopia. Regular and periodic follow-up examinations should be carried out to assess levels of inflammation (i.e., anterior chamber cells and flare, vitreous humor cells and vitreous haze), signs of uncontrolled inflammation (i.e., keratic precipitates and iris nodules), possible complications, and evidence of drug toxicity.^2^ Children should be followed up more closely than adults for evidence of uveitic glaucoma, as glaucomatous optic disc changes can progress very quickly in pediatric patients. Therefore, frequent visual field testing and dilated pupil examination of the optic discs along with optical coherence tomography when needed are strongly recommended. Chronic anterior uveitis patients with no previous systemic disorders (at presentation) should be questioned about the development of joint symptoms due to the fact that arthritis may present after the onset of ocular inflammation in some patients.^21^

Assessment of compliance with the treatment regimen is also critical, because children may need to receive their medications while at school or even apply the topical medications on their own. Compliance issues are common among teenagers that may need to receive a long-term drug therapy. Thus, parents and/or guardians must support and assist with the administration of medications, making sure that doses are not skipped. Considering that pediatric glaucoma can be a chronic, sight-threatening, and stressful condition, support from a team of child psychologists would be beneficial to help the patients and their parents cope with the disease and to improve compliance to treatment and regular follow-up.^21^

## Conclusion

Childhood uveitic glaucoma is one of the most challenging entities in the field of glaucoma, not only because of the unpredictable nature of uveitis but also the difficulty of surgical management due to the risk of failure and complications. Over the last 70 years, a number of operations have been incorporated in the management of childhood glaucoma. Interestingly, most of them have stood the test of time, whereas others have still to prove their efficacy. The fact that there is a wide spectrum of approaches in regard with the management of uveitic glaucoma in children reflects the diversity of its causes and the complexity of its pathogenesis. The challenge of controlling both the inflammatory process and the glaucoma progression together with the absence of controlled trials to facilitate decision-making adds to the perplexity of the situation. The prognosis for childhood uveitic glaucoma has improved substantially over the last decades. However, increasing surgical success rates and reducing complications remains a Gordian knot in modern ophthalmology for specialists who want to ensure a favorable and long-lasting visual outcome for their young patients.

## References

[1] Holland GN, Stiehm ER (2003). Special considerations in the evaluation and management of uveitis in children. Am J Ophthalmol..

[2] Kaur S, Kaushik S, Singh Pandav S (2013). Pediatric Uveitic Glaucoma. J Curr Glaucoma Pract..

[3] Sung VC, Barton K (2004). Management of inflammatory glaucomas. Curr Opin Ophthalmol..

[4] Edelsten C, Reddy MA, Stanford MR, Graham EM (2003). Visual loss associated with pediatric uveitis in English primary and referral centers. Am J Ophthalmol..

[5] Paivonsalo-Hietanen T, Tuominen J, Saari KM (2000). Uveitis in children: population-based study in Finland. Acta Ophthalmol Scand..

[6] Da Mata AP, Foster CS (1999). Ahmed valve and uveitic glaucoma. Int Ophthalmol Clin..

[7] Papadopoulos M, Cable N, Rahi J, Khaw PT; BIG Eye Study Investigators (2007). The British Infantile and Childhood Glaucoma (BIG) Eye Study. Invest Ophthalmol Vis Sci..

[8] Paroli MP, Speranza S, Marino M, Pirraglia MP, Pivetti-Pezzi P (2003). Prognosis of juvenile rheumatoid arthritis-associated uveitis. Eur J Ophthalmol..

[9] Kanda T, Shibata M, Taguchi M, Ishikawa S, Harimoto K, Takeuchi M (2014). Prevalence and aetiology of ocular hypertension in acute and chronic uveitis. Br J Ophthalmol..

[10] Foster CS, Havrlikova K, Baltatzis S, Christen WG, Merayo- Lloves J (2000). Secondary glaucoma in patients with juvenile rheumatoid arthritis-associated iridocyclitis. Acta Ophthalmol Scand..

[11] Heinz C, Koch JM, Zurek-Imhoff B, Heiligenhaus A (2009). Prevalence of uveitic secondary glaucoma and success of nonsurgical treatment in adults and children in a tertiary referral center. Ocul Immunol Inflamm..

[12] Kalogeropoulos D, Sung VC (2018). Pathogenesis of Uveitic Glaucoma. J Curr Glaucoma Pract..

[13] Smith JR (2002). Management of uveitis in pediatric patients: special considerations. Paediatr Drugs..

[14] Giannini EH, Brewer EJ, Kuzmina N, Shaikov A, Maximov A, Vorontsov I, Fink CW, Newman AJ, Cassidy JT, Zemel LS (1992). Methotrexate in resistant juvenile rheumatoid arthritis. Results of the U.S.A.- U.S.S.R. double-blind, placebo-controlled trial. The Pediatric Rheumatology Collaborative Study Group and the Cooperative Children’s Study Group. N Engl J Med..

[15] Wallace CA (1998). The use of methotrexate in childhood rheumatic diseases. Arthritis Rheum..

[16] Tugal-Tutkun I, Havrlikova K, Power WJ, Foster CS (1996). Changing patterns in uveitis of childhood. Ophthalmology..

[17] Quartier P, Baptiste A, Despert V, Allain-Launay E, Koné-Paut I, Belot A, Kodjikian L, Monnet D, Weber M, Elie C, Bodaghi B;, ADJUVITE Study Group (2017). ADJUVITE: a double-blind, randomised, placebo-controlled trial of adalimumab in early onset, chronic, juvenile idiopathic arthritis-associated anterior uveitis. Ann Rheum Dis..

[18] Simonini G, Druce K, Cimaz R, Macfarlane GJ, Jones GT (2014). Current evidence of anti-tumor necrosis factor α treatment efficacy in childhood chronic uveitis: a systematic review and meta-analysis approach of individual drugs. Arthritis Care Res (Hoboken)..

[19] Ramanan AV, Dick AD, Jones AP, McKay A, Williamson PR, Compeyrot- Lacassagne S, Hardwick B, Hickey H, Hughes D, Woo P, Benton D, Edelsten C, Beresford MW;, SYCAMORE Study Group (2017). Adalimumab plus methotrexate for uveitis in juvenile idiopathic arthritis. N Engl J Med..

[20] Heiligenhaus A, Miserocchi E, Heinz C, Gerloni V, Kotaniemi K (2011). Treatment of severe uveitis associated with juvenile idiopathic arthritis with anti-CD20 monoclonal antibody (rituximab). Rheumatology (Oxford)..

[21] Chan NS, Choi J, Cheung CMG (2018). Pediatric Uveitis. Asia Pac J Ophthalmol (Phila)..

[22] Samant M, Medsinge A, Nischal KK (2016). Pediatric Glaucoma: Pharmacotherapeutic Options. Paediatr Drugs..

[23] Passo MS, Palmer EA, Van Buskirk EM (1984). Plasma timolol in glaucoma patients. Ophthalmology..

[24] Schmidtborn F (1998). Systemic side-effects of latanoprost in a child with aniridia and glaucoma. Ophthalmologe..

[25] Babu K, Murthy GJ (2013). Cytomegalovirus anterior uveitis in immunocompetent individuals following topical prostaglandin analogues. J Ophthalmic Inflamm Infect..

[26] Chang L, Ong EL, Bunce C, Brookes J, Papadopoulos M, Khaw PT (2013). A review of the medical treatment of pediatric glaucomas at Moorfields Eye Hospital. J Glaucoma..

[27] Papadopoulos M, Edmunds B, Fenerty C, Khaw PT (2014). Childhood glaucoma surgery in the 21st century. Eye (Lond)..

[28] Papadopoulos M, Edmunds B, Chiang M, Mandal A, Grajewski AL, Khaw PT (2013). Glaucoma Surgery in Children. In: Weinreb RN, Grajewski A, Papadopoulos M, Grigg J, Freedman S, eds Childhood Glaucoma. WGA Consensus Series – 9. Amsterdam: Kugler Publications;.

[29] O’Malley Schotthoefer E, Yanovitch TL, Freedman SF (2008). Aqueous drainage device surgery in refractory pediatric glaucomas: I. Long-term outcomes. J AAPOS..

[30] Christakis PG, Tsai JC, Kalenak JW, Zurakowski D, Cantor LB, Kammer JA, Ahmed II (2013). The Ahmed versus Baerveldt study: three-year treatment outcomes. Ophthalmology..

[31] Kee C (2001). Prevention of early postoperative hypotony by partial ligation of silicone tube in Ahmed glaucoma valve implantation. J Glaucoma..

[32] Englert JA, Freedman SF, Cox TA (1999). The Ahmed valve in refractory pediatric glaucoma. Am J Ophthalmol..

[33] Mandalos A, Sung V (2017). Glaucoma drainage device surgery in children and adults: a comparative study of outcomes and complications. Graefes Arch Clin Exp Ophthalmol..

[34] Kalinina Ayuso V, Scheerlinck LM, de Boer JH (2013). The effect of an Ahmed glaucoma valve implant on corneal endothelial cell density in children with glaucoma secondary to uveitis. Am J Ophthalmol..

[35] Mandalos A, Tailor R, Parmar T, Sung V (2016). The long-term outcomes of glaucoma drainage device in pediatric glaucoma. J Glaucoma..

[36] Chiam PJ, Chen X, Haque MS, Sung VC (2018). Outcome of fixed volume intracameral sodium hyaluronate 1.4% injection for early post‐operative hypotony after Baerveldt glaucoma implant. Clin Exp Ophthalmol..

[37] Beauchamp GR, Parks MM (1979). Filtering surgery in children: barriers to success. Ophthalmology..

[38] Wang Q, Wang J, Fortin E, Hamel P (2016). Trabeculotomy in the Treatment of Pediatric Uveitic Glaucoma. J Glaucoma..

[39] Wiese K, Heiligenhaus A, Heinz C (2014). Trabeculectomy in uveitis associated with juvenile idiopathic arthritis: long-term results in pediatric secondary glaucoma. Ophthalmologe..

[40] Leinonen S, Kotaniemi K, Kivelä T, Majander A (2015). Potential Effect of Tumor Necrosis Factor Inhibitors on Trabeculectomy With Mitomycin C for Patients With Juvenile Idiopathic Arthritis-Related Uveitic Glaucoma: A Retrospective Analysis. JAMA Ophthalmol..

[41] Bohnsack BL, Freedman SF (2013). Surgical outcomes in childhood uveitic glaucoma. Am J Ophthalmol..

[42] Bayraktar S, Koseoglu T (2001). Endoscopic goniotomy with anterior chamber maintainer: Surgical technique and one year results. Ophthalmic Surg Lasers..

[43] Kulkarni SV, Damji KF, Fournier AV, Pan I, Hodge WG (2010). Endoscopic goniotomy: early clinical experience in congenital glaucoma. J Glaucoma..

[44] Papadopoulos M, Khaw PT (2009). Goniotomy and Trabeculotomy. In: Shaarawy T, Sherwood MB, Hitchings RA, Crowston JG, eds. Glaucoma. Philadelphia: Saunders/Elsevier.

[45] Teekhasaenee C, Ritch R (1999). Combined phacoemulsification and goniosynechialysis for uncontrolled chronic angle-closure glaucoma after acute angle-closure glaucoma. Ophthalmology..

[46] Pillunat LE, Erb C, Jünemann AG, Kimmich F (2017). Micro-invasive glaucoma surgery (MIGS): a review of surgical procedures using stents. Clin Ophthalmol..

[47] Caprioli J, Kim JH, Friedman DS, Kiang T, Moster MR, Parrish RK 2nd, Rorer EM, Samuelson T, Tarver ME, Singh K, Eydelman MB (2015). Special commentary: supporting innovation for safe and effective minimally invasive glaucoma surgery: Summary of a Joint Meeting of the American Glaucoma Society and the Food and Drug Administration, Washington, DC, February 26, 2014. Ophthalmology..

[48] Katz LJ, Erb C, Carceller A, Fea AM, Voskanyan L, Wells JM, Giamporcaro JE (2015). Prospective, randomized study of one, two, or three trabecular bypass stents in open-angle glaucoma subjects on topical hypotensive medication. Clin Ophthalmol..

[49] Plager DA, Neely DE (1999). Intermediate-term results of endoscopic diode laser cyclophotocoagulation for pediatric glaucoma. J AAPOS..

[50] Hooper PL, Rao NA, Smith RE (1990). Cataract extraction in uveitis patients. Surv Ophthalmol..

